# GWAS and genetic and phenotypic correlations of plasma metabolites with complete blood count traits in healthy young pigs reveal implications for pig immune response

**DOI:** 10.3389/fmolb.2023.1140375

**Published:** 2023-03-13

**Authors:** E. Dervishi, X. Bai, M. K. Dyck, J. C. S. Harding, F. Fortin, J. C. M. Dekkers, G. Plastow

**Affiliations:** ^1^ Livestock Gentec, Department of Agriculture, Food and Nutritional Science, Faculty of Agricultural, Life and Environmental Sciences, University of Alberta, Edmonton, AB, Canada; ^2^ Department of Large Animal Clinical Sciences, University of Saskatchewan, Saskatoon, SK, Canada; ^3^ Centre de Developpement du porc du Quebec inc (CDPQ), Quebec City, QC, Canada; ^4^ Department of Animal Science, Iowa State University, Ames, IA, United States

**Keywords:** metabolomics, complete blood count, pigs, genetic correlation, GWAS-genome-wide association study

## Abstract

**Introduction:** In this study estimated genetic and phenotypic correlations between fifteen complete blood count (CBC) traits and thirty-three heritable plasma metabolites in young healthy nursery pigs. In addition, it provided an opportunity to identify candidate genes associated with variation in metabolite concentration and their potential association with immune response, disease resilience, and production traits.

**Methods:** The blood samples were collected from healthy young pigs and Nuclear Magnetic Resonance (NMR) was used to quantify plasma metabolites. CBC was determined using the ADVIA_®_ 2120i Hematology System. Genetic correlations of metabolite with CBC traits and single step genome-wide association study (ssGWAS) were estimated using the BLUPF90 programs.

**Results:** Results showed low phenotypic correlation estimates between plasma metabolites and CBC traits. The highest phenotypic correlation was observed between lactic acid and plasma basophil concentration (0.36 ± 0.04; *p* < 0.05). Several significant genetic correlations were found between metabolites and CBC traits. The plasma concentration of proline was genetically positively correlated with hemoglobin concentration (0.94 ± 0.03; *p* < 0.05) and L-tyrosine was negatively correlated with mean corpuscular hemoglobin (MCH; −0.92 ± 0.74; *p* < 0.05). The genomic regions identified in this study only explained a small percentage of the genetic variance of metabolites levels that were genetically correlated with CBC, resilience, and production traits.

**Discussion:** The results of this systems approach suggest that several plasma metabolite phenotypes are phenotypically and genetically correlated with CBC traits, suggesting that they may be potential genetic indicators of immune response following disease challenge. Genomic analysis revealed genes and pathways that might interact to modulate CBC, resilience, and production traits.

## Introduction

Immunity refers to the immune system’s capacity to protect individuals from disease by recognizing and eliminating potentially pathogenic agents, including bacteria, bacterial toxins, viruses, parasites and fungi (Stanfield and Germann, 2008). The earliest line of defence against microbes and pathogens it is provided by innate immunity which is a non-specific response. The principal components of innate immunity include: 1) physical and chemical barriers example: skin, epithelia, the gastrointestinal tract and antimicrobial chemicals produced at epithelial surfaces 2) cellular components include: neutrophils, eosinophils, monocytes, macrophages, dendritic cells, and natural killer (NK) cells; 3) other innate lymphoid cells and blood proteins, including members of the complement system and other mediators of inflammation (Janeway et al., 2001; Stanfield and Germann, 2008). Adaptive immunity is a more sophisticated defense response which uses specific antigens to strategically mount an immune response. Cellular components of adaptive immune system include lymphocytes: B cells and T cells. B cells are made and mature in bone marrow and are responsible for production of antibodies and release them into the blood. T cells migrate from the bone marrow through the blood stream and mature in the thymus (Janeway et al., 2001). T lymphocytes are distinguished by the presence of cell surface molecules CD4 and CD8 and are a major source of cytokines production. T lymphocytes expressing CD4 are known as helper T cells and are subdivided into Th1, Th2, Th17 and inducible regulatory T cells (Paul and Zhu, 2010). The cytokines produced by Th1 and Th2 cells are known as Th1-type cytokines and Th2-type cytokines (Berger, 2000). The cytokines produced by Th1 cells, tend to produce an inflammatory response. Th1 cells mount a host defense against intracellular pathogens such as protozoa, bacteria, and viruses (Butcher and Zhu, 2021) meanwhile Th2 cells participate in different types of allergic disease, but they important in helping to mount a defense against extracellular parasites infections and exposure to venoms (Paul and Zhu, 2010).

Other component of blood such as platelets, possess immune receptors and produce inflammatory molecules (Semple et al., 2011). Platelets also contribute in recruiting leukocytes, principally neutrophils, to the affected site (Kolaczkowska and Kubes, 2013). New findings suggest that red blood cells (RBCs), which develop in bone marrow, are also components of inflammatory responses, as Lam et al. (2021) showed that RBCs serve as critical immune sensors through surface expression of the nucleic acid–sensing Toll-like receptor 9 (TLR9).

In humans, metabolic reprogramming of immune cells has a critical influence on their function (Rodriguez-Coira et al., 2021). For example, sphingolipids are involved in dendritic cell maturation, activation, and migration (Park and Im, 2019). Metabolites such as succinate and citrate have been identified to be involved in innate immune responses by acting as signals in inflammation (Corcoran and O’Neill, 2016). In addition, short chain fatty acids (SCFA) and biogenic amines, enhance dendritic cell regulatory activity (Tan et al., 2014).

Therefore, understanding the impact of different metabolites on metabolic reprogramming of immune cells is important to improve diagnosis, prognosis, and therapeutic personalized medicine strategies in humans (Rodriguez-Coira et al., 2021). In this regard, pigs serve in biomedical research as an animal model because of their similarity with human physiology. Therefore, understanding the relationship of metabolites and immune response cells in pigs can offer insights for human physiology and immune response.

In the last decade, metabolomics has been used to discover biomarkers of disease in different livestock species as well as in animal genetic studies because it provides the potential to identify new phenotypes or traits which can be used to select for more efficient and resilient animals (Montgomery et al., 2009; Widmann et al., 2013; Karisa et al., 2014; Dervishi et al., 2018a; Dervishi et al., 2018b; Carmelo et al., 2020; Li et al., 2020). It is therefore important to understand the genetic architecture of these potential new phenotypes and their relationship with immune response.

The importance of health and the immune system in relation to productivity in pigs facing disease challenge has been described (Bai et al., 2020; Cheng et al., 2020). Bai et al. (2020) reported that pigs classified as resilient initiated a faster adaptive immune response and recovered earlier following infection showing a greater increase in lymphocyte concentration in blood collected 2- weeks before and 2- weeks after the disease challenge, compared to susceptible pigs. In addition, Bai et al. (2020) reported estimates of heritability of CBC traits and the phenotypic and genetic correlations of CBC traits with growth rate and veterinary treatment rate. Estimates of the heritability of 44 metabolites on young healthy pigs along with estimates of the phenotypic and genetic parameters of plasma metabolite concentration with subsequent performance, disease resilience, and carcass traits under the same natural disease challenge described by Bai et al. (2020) was reported by Dervishi et al. (2021).

The present study is part of a larger project which has identification of predictors of disease resilience in young healthy pigs prior to a disease challenge as its main objective. For this purpose, a natural polymicrobial disease challenge model (Cheng et al., 2020; Bai et al., 2020) was established and numerous samples and traits were collected before and after challenge, including complete blood count traits (CBC), average daily gain (ADG), feed intake and feed intake duration (ADFI and ADFD), number of individual health treatments (nTRT), mortality, residual feed intake (RFI), and feed conversion ratio (FCR) (Dervishi et al., 2021; Bai et al., 2020; Cheng et al., 2020).

The purpose of this study was to estimate the phenotypic and genetic parameters between CBC traits and plasma metabolite concentrations in plasma samples collected on young healthy pigs, prior to the disease challenge. Furthermore, we attempted to identify genomic regions that control the genetic variance of metabolites that are genetically correlated with CBC, resilience, and production traits.

## Material and methods

### Ethics statement

The experiment was carried out in accordance with the Canadian Council on Animal Care guidelines (CCAC, 1993) and (Kilkenny et al., 2010) Animal Research: Reporting of *In Vivo* Experiments guidelines ARRIVE; https://arriveguidelines.org; (Percie du Sert et al., 2020). The animal experiments were performed with the approval of the Animal Protection Committee of the Centre de Recherche en Sciences Animales de Deschambault (15PO283) and the Animal Care and Use Committee at the University of Alberta (AUP00002227).

### Experimental design

All the details of the polymicrobial challenge together with phenotypes/traits that were collected were described by Putz et al. (2019), Cheng et al. (2020) and Bai et al. (2020). Briefly, healthy F1 crossbred (Landrace × Yorkshire) castrated male weaned pigs were provided in rotation by seven genetic suppliers, all members of the PigGen Canada research consortium. Each batch consisted of approximately 65 or 75 pigs from a healthy multiplier farm from one of the genetic suppliers (Bai et al., 2020). All weaned pigs arrived at an average age of 21 days and were housed in a quarantine nursery (Supplementary Figure S1). At approximately 40 days of age, pigs were transferred to the test station late nursery (challenge nursery) and exposed to multiple pathogens through contact with the previous batch that entered 3-weeks prior. Pigs were sent for slaughter when they reached the slaughter weight of 130 kg, at approximately 181 days of age (Bai et al., 2020).

Jugular blood was collected into K2 ethylenediaminetetraacetic acid (EDTA) tubes (BD Vacutainer, Blood Collection Tubes, United States) from all pigs in the quarantine nursery 5 days post-arrival at average 26 days of age (Bai et al., 2020) for CBC and metabolomics analysis. Complete blood count, number of treatments, mortality, growth rate, feed intake and feed efficiency, were collected from a total of 3,205 F1 crossbred pigs, either when sent to slaughter or euthanized at humane end points specified for animal welfare.

All animals were genotyped using a 650 k Affymetrix Axiom Porcine Genotyping Array at Delta Genomics (Edmonton AB, Canada). Raw Affymetrix SNP data were processed by Delta Genomics, separately for each cycle, using the Axiom Analysis Suite. All the details of genotyping and quality control have been previously described (Putz et al., 2019; Bai et al., 2020; Cheng et al., 2020). A total of 417,443 SNPs for 3,205 pigs remained after quality control and were used for analysis (Dervishi et al., 2021).

### Complete blood count and metabolomics traits

In this study we used CBC and metabolomics data obtained from blood samples collected in the quarantine nursery from 968 pigs. Details of the CBC and metabolomic data have been described by Bai et al. (2020) and Dervishi et al. (2021) respectively. CBC analysis was performed using the ADVIA^®^ 2120i Hematology System (Siemens Healthineers, Erlangen, Germany). A total of 15 CBC traits were included: concentration of total white blood cell concentration (WBC, 10^3^/μL), neutrophils (NEU, 10^3^/μL), lymphocytes (LYM, 10^3^/μL), monocytes (MONO, 10^3^/μL), eosinophils (EOS, 10^3^/μL), basophils (BASO, 10^3^/μL), red blood cells (RBC, 10^6^/μL), hemoglobin (HGB, g/L), hematocrit (HCT, %), mean corpuscular volume (MCV, fL), mean corpuscular hemoglobin (MCH, pg), mean corpuscular hemoglobin concentration (MCHC, g/L), red blood cell distribution width (RDW, %), platelet concentration (PLT, 10^3^/μL), and mean platelet volume (MPV, fL). Descriptive statistics including mean and standard deviation values of CBC traits by batch, for the pigs included in the analysis, are shown in Supplementary Table S1.

Details of metabolomics analysis are described in Dervishi et al. (2021). In order to remove plasma macromolecules, samples were thawed on ice and a deproteinization step, involving ultra-filtration was performed (Psychogios et al., 2011). Prior to filtration process, a 3 kDa cut-off centrifugal filter units (Amicon Microcon YM-3), were rinsed five times each with 0.5 mL of H_2_O and centrifuged (10,000 rpm for 10 min) to remove residual glycerol bound to the filter membranes. To remove macromolecules from the sample (primarily protein and lipoproteins), aliquots of each sample were transferred into the centrifuge filter devices and spun at 10,000 rpm during 20 min (Dervishi et al., 2021). After collecting the filtrates, the volumes for each sample were recorded. If the total volume of the sample was under 250 µL an appropriate amount of 150 mM KH_2_PO_4_ buffer (pH 7) was added and the dilution factor was annotated and metabolite concentrations were corrected in the subsequent analysis. Thereafter, 46.5 µL of a standard buffer solution (54% D_2_O:46% 1.75 mM KH_2_PO_4_ pH 7.0 v/v containing 5.84 mM DSS (2,2-dimethyl-2-silcepentane-5-sulphonate), 5.84 mM 2-chloropyrimidine-5 carboxylate, and 0.1% NaN_3_ in H_2_O) was added to the sample (Dervishi et al., 2021). After preparation step, plasma samples (250 µL) were transferred in 3 mm SampleJet NMR tubes for spectral analysis and ^1^H-NMR spectra were collected on a 700 MHz Avance III (Bruker) spectrometer equipped with a 5 mm HCN Z-gradient pulsed-field gradient (PFG) cryoprobe (Dervishi et al., 2021). ^1^H-NMR spectra were acquired at 25°C using the first transient of the NOESY pre-saturation pulse sequence (noesy1dpr), chosen for its high degree of quantitative accuracy (Saude et al., 2006). All free induction decays (FID’s) were zero-filled to 250 K data points. The singlet produced by the DSS methyl groups was used as an internal standard for chemical shift referencing (set to 0 ppm). The quantification all ^1^H-NMR spectra were processed and analyzed using an in-house version of the MAGMET automated analysis software package using a custom metabolite library (Dervishi et al., 2021). MAGMET allows for qualitative and quantitative analysis of an NMR spectrum by automatically fitting spectral signatures from an internal database to the spectrum. This fitting procedure provides absolute concentration accuracy of 90% or better (Ravanbakhsh et al., 2015). An NMR spectroscopist inspected all spectra to minimize compound misidentification and misquantification (Dervishi et al., 2021). A representative NMR spectrum with assignments it is provided in Supplementary Figure S2.

Forty-four metabolites were quantified: amino acids (AAs), short chain fatty acids (SCFA), sugars, alcohols, organic acids, amines, TCA cycle intermediates and urea cycle intermediates (Dervishi et al., 2021). In addition, two indexes were calculated: 1) ketogenic amino acids (ketoAA), calculated as the sum of L-lysine and L-leucine and 2) the sum of branched amino acids (BCAA) that was calculated as the sum of L-leucine, L-isoleucine and L-valine (Dervishi et al., 2021). Dervishi et al. (2021) reported that the concentrations of 33 metabolites were heritable; in the present study we only considered these for estimation of genetic correlations between CBC and metabolite traits and subsequent GWAS. The observations for leukocyte count (white blood cells) and concentrations of 2-hydroxybutyrate and L-alpha aminobutyric acid were not normally distributed and, therefore, were log-transformed before statistical analyses.

### Estimation of genetic correlations

Genetic correlations of metabolite with CBC traits were estimated using AIREMLF90 of the BLUPF90 programs (Misztal et al., 2002), with the following bivariate mixed linear model described by Dervishi et al. (2021):

$$\Upsilon_{ijk}={Batch}_{i}+{Age}_{ijk}+{Pen}_{j}+{Litter}_{ijk}+u_{ijk}+e_{ijk}$$

where *Υ*_*ijk*_ is the phenotype for the trait (metabolite, CBC) for one of the 968 analyzed pigs; *Batch*_*i*_ is the fixed effect (*i* = 1, …, 15); *Age*_*ijk*_ is the covariate of age when the pig entered the quarantine nursery; *Pen*_*j*_ is the random effect of nursery pen by batch, with *Pen*_*j*_ *∼* N (0, σ^2^_P_), where σ^2^_P_ is pen variance; *Litter*_*ijk*_ is the common environmental effect associated with litter, with *Litter*_*ijk*_ *∼* N (0, σ^2^_L_), where σ^2^_L_ is the litter environmental variance; *u*_*ijk*_ is the random additive genetic effect, with the vector ***u*** ∼ N (**0**, **G**σ^2^_A_), where **G** is the genomic relationship matrix and σ^2^_A_ is the additive genetic variance; and *e*_*ijk*_ is the residual effect, with *e*_*ijk*_ ∼ N (0, σ^2^_e_), where σ^2^_e_ is the residual variance (Dervishi et al., 2021). Environmental enrichment was included as fixed effect for 3-methyl 2-oxovaleric acid and amino acids L-ornithine, L-leucine, L-valine, L-asparagine because it was previously found to be significant (*p* ≤ 0.05; Dervishi et al., 2021). The genomic relationship matrix, **G**, was created using the software preGSf90 (Misztal et al., 2002) and the method described by VanRaden (2008). Matrix **G**, was first created separately for pigs from each of the seven companies and thereafter combined into one **G** matrix. In order to focus on the within-company variance components, genetic relationships between companies was set to zero as described by Cheng et al. (2020). In addition, we estimated phenotypic and genetic correlations among metabolites that belong to the same pathway. Genetic correlations between two traits were estimated as the estimate of the genetic covariance from the bivariate analysis divided by the product of the genetic standard deviations for the two traits. A likelihood ratio test with 1 degree of freedom was used to determine the significance of correlation estimates (Dervishi et al., 2021).

### GWAS and functional analyses

Phenotypic data (concentration of 33 plasma metabolites) were available for 968 F1 crossbred pigs and genotypic data were available for 3,205 F1 crossbred pigs. The complete pedigree for 3,205 pigs was unavailable due to the use of pooled semen in some batches (Bai et al., 2020), however dam information was available for 3,194 pigs and sire information was available for 1,138 pigs.

Single step genome-wide association study (ssGWAS), was performed using the programs of BLUPF90 software family (Misztal et al., 2002; Wang et al., 2014), modified to account for genomic information (Geiger et al., 2016). Single step GWAS integrates pedigree and genomic data in a single step (**H** matrix; Geiger et al., 2016). The inverse of **H** matrix needed for mixed model equations is given by:

$$H^{-1}=A^{-1}+\left[\begin{array}{l} 0\;\;\;\;\;0\;\\ 0\;G^{-1}-{A_{22}}^{-1} \end{array}\right]$$

where **A** ^−1^ is the inverse of the numerator relationship matrix; **A**_22_^−1^ is the inverse of the pedigree relationship matrix; and **G** ^−**1**^ is the inverse of the genomic relationship matrix. The genomic relationship matrix (**G**) was constructed as: G = ZDZ^′^q (VanRaden, 2008); where Z is the incidence matrix containing genotypes (aa = 0, Aa = 1 and AA = 2) adjusted for allele frequency, **D** is a diagonal matrix of weights for SNP markers (initially D = I), and q is a weighting factor. The weighting factor was as in Vitezica et al. (2011), ensuring that the average diagonal in G is close to that of A_22_. The genomic estimated breeding value (GEBV, **â**_**g**_) was calculated by ssGBLUP and the solutions of the SNP effects (**û**) were obtained using the AIREMLF90 (Wang et al., 2012) algorithm. Briefly, on the first step, set **D** = **I**, which gives a weight of 1 to all SNP, and **G** = ZDZ^′^q where **G** is the genomic relationship matrix. On the second step we estimated **â**_**g**_, which were converted to SNP effects: **û** = λDZ’G−1 â_g_, where â_g_ is the GEBV of genotyped animals. This process was run for one iteration.

In this study, SNPs located within 0.5 Mb were grouped as a single window, and the percentage of genetic variance (GV) explained by each window was calculated using the postGSF90 module as: [Var(a_i_)/σ^2^a] x 100%, where a_i_ is the genetic value of the *i*th SNP window and σ^2^a is the additive GV (Wang et al., 2014). The results of GWAS were reported as the proportion of the genetic variance explained by non-overlapping genomic windows (0.5 Mb). The windows that explained equal to or greater than 0.5% of the genetic variance from ssGWAS were considered as QTL regions (Hong et al., 2020). The model for GWAS was as follow:

Y=Xb+Wc+Ul+Za+e

where ***Y*** is the vector of the phenotypes (metabolite concentration); ***b*** is the vector of fixed effects (batch and covariate of age when the pig entered the quarantine nursery); ***X*** is the incidence matrix relating observations to the fixed effects, ***c*** is the vector for the random pen effect; ***W*** is the incidence matrix of the random pen effect; ***l*** is the vector of for the random litter effect; ***U*** is the incidence matrix of the random litter effects; **a** is the vector of direct additive genetic effects; **Z** is the incidence matrix of random animal effects; and **e** is a vector of the random residuals.

Batches were nested within farms and coded uniquely; therefore population stratification was accounted for in the association analysis by fitting batch as the fixed effect in the model (Bai et al., 2020).

The identification of candidate genes was performed using *Sus scrofa* genome version 11.1 in BioMart (http://uswest.ensembl.org/biomart/martview/73240e1280d6d0c946725fde6eb27af9). Genecards (https://www.genecards.org/) was used to investigate the gene function based on orthologous genes of humans. Candidate genes were also compared with the information about QTLs reported in QTLdb (Hu et al., 2016; http://www.animalgenome.org/cgi-bin/QTLdb/index).

The Metscape plugin (Karnovsky et al., 2012) in Cytoscape 3.8.2 (Shannon et al., 2003) was used to explore and visualize the biochemical pathway that metabolites are involved in. Metabolites that had significant (*p* ≤ 0.05) genetic correlations with traits of interest (example: NEU, MONO, EOS, BASO, LYM, ADG) were used for network visualization. In order to generate a gene-compound network, a file containing the list of KEGG elements was loaded into Metscape following instructions provided by (Karnovsky et al., 2012). In a gene-compound network, genes and metabolites are represented as nodes and reactions are represented as edges. A compound node with an outgoing edge is a substrate, while a compound node with an incoming edge is the product of a specific biochemical reaction (Dervishi et al., 2021).

## Results

### Phenotypic correlations

In general, estimates of phenotypic correlations of the metabolites with CBC were small (Supplementary Figure S3). The estimate of greatest magnitude was between L-glutamic acid and PLT. L-lactic acid was positively correlated with BASO, WBC, NEU, EOS, and MPV, while it showed negative phenotypic correlation with MCHC (*p* < 0.05). Pyruvic acid showed positive phenotypic correlation estimates with HCT, MCV, MCH (*p* < 0.05), and negative correlations with PLT, RDW, and MPV (*p* < 0.05). D-glucose showed negative phenotypic correlation estimates with NEU, BASO, EOS, MONO, LYM, and MPV. Furthermore, L-alanine showed negative phenotypic correlation with MCHC (Supplementary Table S2; *p* < 0.05). Finally, oxoglutarate was positively correlated with HGB (*p* < 0.05). A scatter plot showing the negative correlations between pyruvic acid, D-glucose and CBC traits is shown in Supplementary Figure S4.

The phenotypic correlation between metabolites that are involved in the same pathway (betaine, dimethylglycine, L-glycine, L-glutamic acid, L-serine, oxoglutarate, L-glutamine and methionine) are shown in Table 3. The highest significant positive correlation was observed between oxoglutarate an L-glutamic acid.

### Genetic correlations

Overall, estimates of genetic correlations of the metabolites with CBC traits were larger than phenotypic correlations (Tables 1, 2) but had larger SE. Table 1 shows the estimates of genetic correlation between metabolites and white blood cell traits. Some metabolites showed significant correlation estimates (*p* < 0.05) with white blood cell traits. For example, a high negative genetic correlation between NEU and plasma L-lysine was observed (*p* < 0.05). L-glutamine was positively correlated with NEU (*p* < 0.05) and L-aspartate was positively correlated with MONO (*p* < 0.05). 2-hydroxybutyrate, 3-methyl-2-oxovaleric acid and L-alpha aminobutyric acid had positive genetic correlation estimates with EOS, while dimethylglycine was negatively correlated with EOS concentration (*p* < 0.05). Positive genetic correlations were estimated for WBC with the amino acids L-asparagine and L-glutamic acid (*p* < 0.05). Lymphocyte concentration was positively correlated with isobutyric acid and L-histidine (*p* < 0.05), while LYM was negatively correlated with hypoxanthine and L-ornithine (*p* < 0.05; Table 1).

**TABLE 1:** Estimates and standard errors (SE) of genetic correlations of metabolite correlations^a^ with white blood cells traits^b^.

Metabolite	NEU	MONO	EOS	BASO	LYM	WBC
2-Hydroxybutyrate	0.07 (0.73)	−0.21 (0.70)	0.86 (0.78)**	0.51 (0.80)	0.31 (0.79)	0.11 (0.02)
3-Methyl-2-oxovaleric acid	−0.04 (0.63)	0.41 (1.0)	0.68 (0.05)*	0.37 (2.32)	−0.45 (0.85)	−0.30 (0.79)
Betaine	−0.25 (0.27)	0.06 (0.28)	0.06 (0.20)	−0.12 (0.9)	−0.013 (0.23)	−0.12 (0.29)
Citric acid	-	0.22 (0.34)	-	−0.45 (0.78)	-	−0.01 (0.37)
Creatinine	−0.09 (0.27)	−0.08 (0.26)	−0.08 (0.20)	0.35 (0.78)	0.12 (0.22)	−0.09 (0.30)
D-glucose	−0.28 (0.34)	−0.29 (0.35)	0.04 (0.3)	−0.65 (0.77)	−0.006 (0.31)	-
Dimethylglycine	−1.06 (1.48)	0.20 (0.40)	−0.37 (0.20)*	−0.5 (0.80)	0.59 (0.60)	−0.29 (0.29)
Hypoxanthine	0.11 (0.28)	−0.22 (0.29)	0.12 (0.18)	−0.33 (3.81)	−0.42 (0.23)*	−0.02 (0.29)
Isobutyric acid	0.10 (0.42)	−0.06 (0.45)	0.043 (0.06)	0.85 (1.32)	0.69 (0.35)***	0.37 (0.40)
L-alanine	0.45 (0.52)	0.02 (0.5)	−0.72 (1.05)	−0.06 (0.04)	-	0.12 (0.47)
L-alpha-aminobutyric acid	0.56 (0.44)	0.40 (0.36)^X^	0.75 (0.51)*	0.70 (0.87)	1.0 (0.90)*	0.86 (0.86)
L-asparagine	0.41 (0.54)	−0.01 (0.51)	0.19 (0.40)	0.73 (1.54)	0.56 (0.69)	0.74 (0.58)*
L-aspartate	0.34 (0.85)	0.66 (0.58)*	0.09 (0.30)	0.86 (1.89)	0.10 (0.36)	0.15 (0.50)
L-glutamine	0.75 (0.30)***	−0.08 (0.25)	0.11 (0.18)	0.56 (0.89)	0.06 (0.20)	0.51 (0.29)*
L-glutamic acid	-	0.01 (0.27)	0.27 (0.12)	0.61 (0.92)	−0.0035 (0.2)	0.41 (0.06)**
L-glycine	0.02 (0.26)	0.02 (0.35)	−0.14 (0.20)	0.06 (0.64)	0.26 (0.23)	−0.04 (0.29)
L-histidine	−0.006 (0.38)	−0.04 (0.35)	0.01 (0.02)	0.67 (3.09)	0.33 (0.29)*	-
L-isoleucine	−0.08 (0.86)	0.11 (6.0)	0.50 (0.69)	0.33 (1.18)	−0.58 (0.69)	0.11 (0.02)
L-Lactic acid	0.19 (0.42)	0.0003 (0.4)	0.13 (0.26)	0.57 (0.68)	−0.01 (0.30)	−0.17 (0.50)
L-lysine	−0.81 (0.4)*	−0.17 (0.32)	0.19 (0.24)	−0.02 (0.72)	0.16 (0.26)	−0.32 (0.56)
L-leucine	−0.09 (0.46)	0.77 (0.81)	0.27 (0.30)	0.43 (0.95)	−0.07 (0.36)	−0.26 (0.56)
L-methionine	0.22 (0.45)	−0.51 (0.52)	0.39 (0.29)^X^	−0.08 (0.1)	−0.26 (0.36)	-
L-ornithine	−0.34 (0.33)	-	−0.10 (0.21)	−0.39 (0.64)	−0.42 (0.21)*	-
L-phenylalanine	−0.56 (0.95)	0.79 (0.51)	-	0.43 (1.08)	−0.15 (0.49)	−0.51 (1.35)
L-proline	0.34 (0.69)	0.004 (1.01)	−0.04 (0.01)	0.45 (1.03)	-	0
L-serine	−0.23 (0.48)	−0.26 (0.44)	−0.04 (0.31)	−0.19 (0.96)	0.37 (0.43)	0.006 (0.4)
Oxoglutarate	-	−0.32 (0.55)	0.25 (0.20)	0.56 (0.94)	−0.20 (0.1)	−0.33 (0.71)
Pyruvic acid	−0.03 (0.32)	0.18 (0.33)	0.13 (0.23)	0.70 (0.97)	0.36 (0.24)^X^	0.42 (0.36)
L-threonine	−0.49 (1.57)	−0.24 (0.86)	−0.75 (1.12)	−0.03 (1.37)	0.07 (0.52)	−0.31 (0.65)
L-tyrosine	-	0.12 (0.93)	−0.1 (0.33)	0.28 (1.0)	−0.16 (0.40)	−0.15 (0.97)
L-valine	−0.14 (0.55)	0.03 (0.54)	0.08 (0.37)	0.52 (0.81)	−0.06 (0.41)	−0.06 (0.67)
BCAA	−0.12 (0.48)	-	0.22 (0.34)	0.45 (0.81)	−0.18 (0.39)	−0.25 (1.24)
KetoAA	−0.81 (0.59)*	0.21 (1.27)	0.25 (0.27)	0.10 (0.87)	0.12 (0.30)	−0.33 (0.71)

^a^ BCAA, Branched-chain amino acid index was calculated as the sum of L-leucine, L-isoleucine and L-valine and ketoAA, ketogenic amino acids was calculated as the sum of L-lysine and L-leucine.

^b^ NEU, neutrophil concentration (10^3^/μL); MONO, monocyte concentration (10^3^/μL); EOS, eosinophil; BASO, basophil concentration (10^3^/μL); LYM, lymphocyte concentration (10^3^/μL); WBC, total white blood cell concentration (10^3^/μL).

Significance of the genetic correlations are highlighted in bold based on the likelihood ratio test and indicated as ***, **, *, ^X^, corresponding to *p* < 0.001, *p* < 0.01 and *p* ≤ 0.05, and 0.05 < *p* < 0.10 respectively; “-” indicates not estimable.

**TABLE 2:** Estimates and standard errors (SE) of genetic correlations of metabolite concentrations^a^ with red blood cell and platelet traits^b^.

Metabolite	RBC	HGB	HCT	MCV	MCH	MCHC	RDW	PLT	MPV
2-Hydroxybutyrate	0.05 (0.41)	0.05 (1.27)	0.21 (1.27)	0.03 (0.4)	−0.25 (0.37)	0.07 (0.7)	−0.83 (0.03)	−0.78 (0.83)	−0.2 (0.42)
3-Methyl-2-oxovaleric acid	0.29 (0.64)	0.62 (2.06)	0.51 (2.0)	−0.36 (0.45)	−0.48 (0.07)	−0.08 (0.74)	0.43 (0.08)	−1.02 (2.25)	0.52 (0.80)
Betaine	0.26 (0.25)	0.37 (0.50)	0.68 (1.09)	−0.07 (0.20)	0.03 (0.19)	−0.01 (0.31)	0.45 (0.30)	*0.48 (0.46)*	−0.04 (0.26)
Citric acid	0.007 (0.26)	−0.17 (0.57)	0.37 (0.58)	0.11 (0.20)	−0.18 (0.21)	−0.11 (0.4)	−0.78 (1.48)	0.13 (0.42)	−0.14 (0.26)
Creatinine	0.04 (0.23)	0.19 (0.47)	−0.12 (0.68)	0.11 (0.18)	0.19 (0.18)	0.04 (0.28)	−0.75 (0.90)	−0.08 (0.34)	−0.06 (0.33)
D-glucose	−0.05 (0.30)	−0.56 (0.72)	−0.25 (0.58)	−0.11 (0.26)	−0.06 (0.27)	0.33 (0.54)	−0.22 (1.15)	-	0.02 (0.34)
Dimethylglycine	−0.03 (0.24)	0.07 (0.4)	0.21 (0.49)	0.08 (0.18)	0.18 (0.18)	−0.17 (0.27)	0.50 (0.10)	-	0.17 (0.25)
Hypoxanthine	0.21 (0.22)	0.48 (0.52)	0.63 (0.49)*	0.21 (0.16)	0.08 (0.28)	0.08 (0.28)	−0.30 (0.76)	−0.42 (0.4)	−0.02 (0.23)
Isobutyric acid	−0.20 (0.35)	0.27 (0.66)	−0.13 (0.71)	0.17 (0.31)	0.18 (0.34)	0.49 (0.52)	−0.76 (0.04)^X^	−0.44 (0.90)	−0.17 (0.36)
L-alanine	0.54 (0.48)^X^	0.44 (1.06)	0.17 (0.06)	0.11 (0.28)	0.09 (0.28)	−0.36 (0.43)	−0.64 (0.05)*	0.98 (0.05)**	0.07 (0.39)
L-alpha-aminobutyric acid	0.14 (0.32)	0.24 (0.67)	0.56 (0.69)	0.26 (0.31)	0.06 (0.30)	−0.07 (0.37)	−0.98 (0.03)**	−0.85 (1.54)	−0.13 (0.33)
L-asparagine	−0.02 (0.55)	0.07 (1.33)	0.29 (0.92)	0.32 (0.52)	0.40 (0.59)	0.2 (0.81)	−0.10 (1.41)	−0.30 (2.17)	−0.42 (0.91)
L-aspartate	0.05 (0.40)	−0.36 (0.59)	−0.35 (0.73)	−0.24 (0.29)	−0.16 (0.38)	0.25 (0.44)	−0.52 (1.23)	−0.54 (0.73)	0.01 (0.04)
L-glutamine	0.07 (0.22)	0.37 (0.62)	0.36 (0.60)	0.18 (0.17)	0.16 (0.18)	0.16 (0.28)	−0.32 (0.88)	−0.56 (0.55)	−0.21 (0.23)
L-glutamic acid	0.03 (0.23)	0.03 (0.39)	−0.08 (0.48)	−0.12 (0.16)	−0.01 (0.17)	0.22 (0.26)	−0.42 (0.06)	−0.47 (0.07)	−0.10 (0.23)
L-glycine	0	0.09 (0.59)	0.27 (0.59)	0.13 (0.18)	0.45 (0.17)**	0.26 (0.29)	−0.19 (0.94)	0.16 (0.45)	0.23 (0.25)
L-histidine	−0.02 (030)	0.19 (0.56)	−0.73 (0.88)	−0.20 (0.24)	0.17 (0.23)	0.37 (0.34)	−0.36 (0.80)	−0.72 (1.05)	−0.21 (0.39)
L-isoleucine	0.55 (0.75)	0.97 (0.25)^X^	0.82 (1.78)	−0.26 (0.50)	−0.34 (0.52)	−0.03 (0.78)	0.08 (1.35)	−0.68 (1.11)	0.33 (0.77)
L-Lactic acid	0.61 (0.71)	0.93 (1)	0.67 (0.85)	−0.002 (0.38)	−0.02 (0.39)	−0.26 (0.54)	−0.25 (2.09)	-	−0.38 (0.61)
L-lysine	0.15 (0.32)	0.04 (0.59)	−0.02 (0.71)	−0.27 (0.25)	−0.19 (0.24)	0.29 (0.33)	−0.65 (0.05)	−0.24 (0.44)	−0.17 (0.29)
L-leucine	0.42 (0.43)	0.82 (0.90)	0.35 (0.79)	−0.23 (0.32)	−0.12 (0.33)	−0.38 (0.78)	−0.21 (1.45)	−0.14 (0.78)	0.17 (0.48)
L-methionine	0.13 (0.35)	−0.27 (3.57)	0.018 (0.72)	−0.10 (0.39)	−0.33 (0.25)	−0.49 (0.45)	0.75 (0.70)	−0.56 (0.83)	−0.56 (0.41)*
L-ornithine	−0.11 (0.25)	−0.07 (0.46)	−0.19 (0.62)	*−0.34 (0.21)*	−0.24 (0.22)	−0.07 (0.11)	−0.23 (1.52)	−0.09 (0.40)	0.36 (0.28)
L-phenylalanine	0	−0.14 (1.38)	−0.41 (0.95)	−0.32 (0.47)	−0.34 (0.36)	−0.01 (0.51)	−0.03 (1.25)	−0.49 (0.66)	0.18 (0.60)
L-proline	0.90 (0.97)	0.94 (0.03)*	0.70 (0.04)**	0.18 (0.32)	0.08 (0.57)	−0.39 (0.30)	−0.68 (0.06)	0.02 (0.04)	0.20 (0.66)
L-serine	0.01 (0.35)	−0.04 (0.68)	0.6 (1.13)	0.05 (0.27)	0.17 (0.35)	0.27 (0.49)	0.79 (0.70)	−0.007 (0.71)	0.18 (0.38)
Oxoglutarate	0.08 (0.24)	0.12 (0.64)	0.32 (0.61)	−0.007 (0.19)	0.18 (0.19)	0.31 (0.28)	−0.82 (0.10)^X^	−0.54 (0.48)^X^	−0.08 (0.28)
Pyruvic acid	0.18 (0.31)	0.52 (0.83)	0.57 (0.64)	0.07 (0.74)	0.01 (0.26)	−0.47 (0.63)	−0.75 (0.29)	−0.12 (0.51)	−0.06 (0.28)
L-threonine	−0.06 (0.56)	0.01 (0.96)	0.26 (1.63)	0.09 (0.50)	0.04 (0.48)	−0.1 (0.84)	0.92 (0.16)	0.62 (1.63)	−0.12 (0.55)
L-tyrosine	0.44 (0.45)	−0.10 (1.14)	0.37 (0.95)	−0.73 (0.68)*	−0.92(0.74)**	−0.42 (0.54)	0.66 (1.02)	−0.20 (0.52)	−0.22 (0.46)
L-valine	0.11 (0.49)	0.48 (1.25)	0.16 (0.98)	−0.15 (0.67)	−0.02 (0.63)	0	-	0.26 (0.97)	0.13 (0.59)
BCAA	0.33 (0.46)	0.69 (0.78)	0.34 (0.99)	−0.21 (0.32)	−0.12 (0.31)	−10 (0.46)	−0.04 (1.32)	−0.12 (0.93)	0.16 (0.42)
KetoAA	0.24 (0.33)	0.24 (0.91)	0.07 (0.65)	−0.33 (0.32)	−0.22 (0.39)	0.17 (0.58)	−0.68 (0.05)^X^	−0.27 (0.63)	−0.16 (0.36)

^a^ BCAA, branched-chain amino acid index was calculated as the sum of L-leucine, L-isoleucine and L-valine; ketoAA, ketogenic amino acids was calculated as the sum of L-lysine and L-leucine.

^b^ RBC, red blood cell concentration (10^6^/μL); HGB, hemoglobin concentration (g/L); HCT, hematocrit (%); MCV, mean corpuscular volume (fL); MCH, mean corpuscular hemoglobin (pg); MCHC, mean corpuscular hemoglobin concentration (g/L); RDW, red blood cell distribution width (%). PLT, platelet concentration (10^3^/μL) and MPV, mean platelet volume (fL).

Significance of the genetic correlations are highlighted in bold based on the likelihood ratio test and indicated as ***, **, *, ^X^, corresponding to *p* < 0.001, *p* < 0.01 and *p* ≤ 0.05, and 0.05 < *p* < 0.10 respectively; “-” indicates not estimable.

**FIGURE 1:**
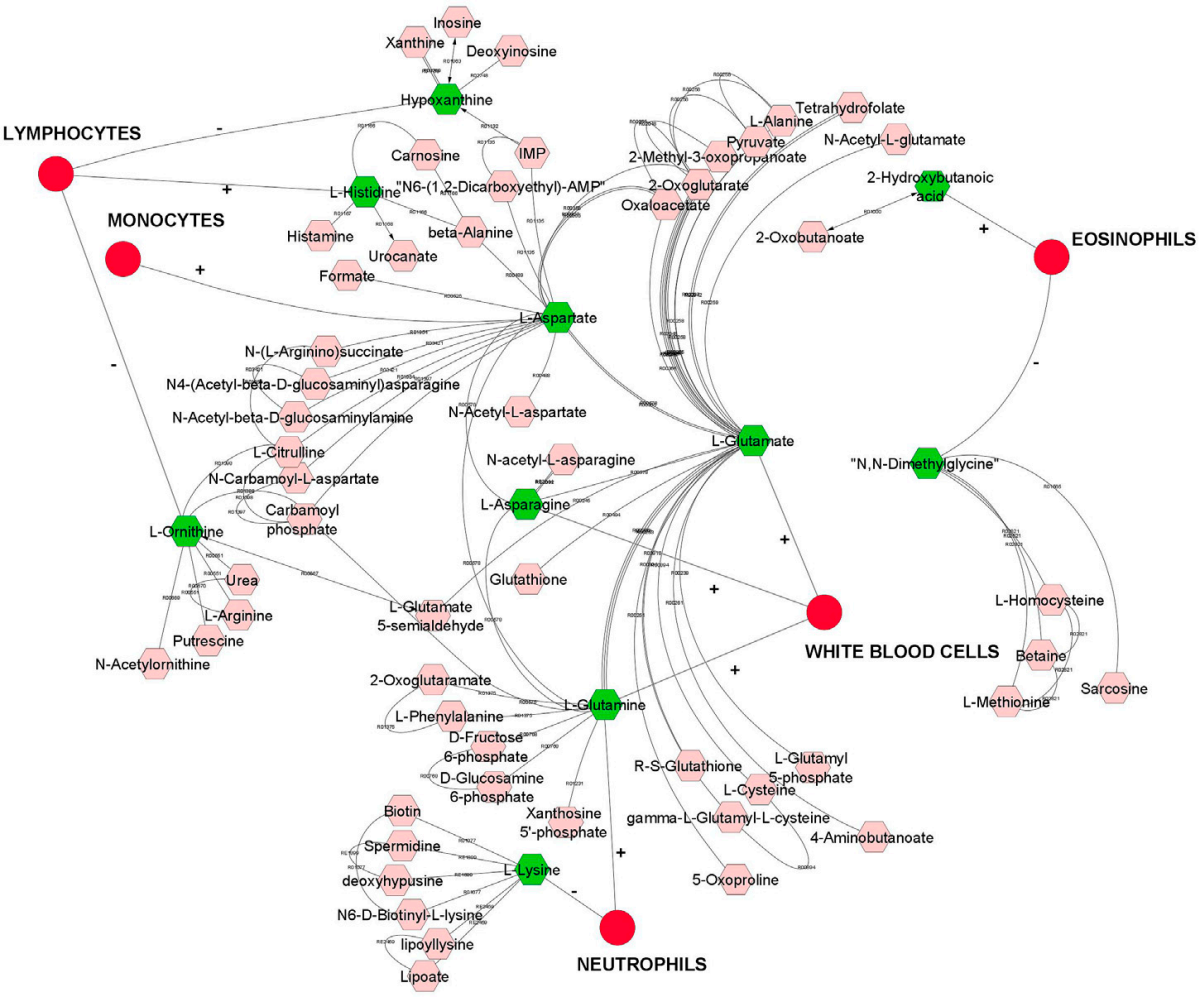
A compound network of metabolites that are significantly (*p* < 0.05) genetically correlated with leukocytes (white blood cells). Input metabolites are shown in green color and chemical reactions are represented as edges. Positive or negative sign on the edges represent positive or negative genetic correlation.

Overall, we found a higher number of significant genetic correlation between metabolites and leukocytes than RBC or platelets traits. Therefore, metabolites that were genetically correlated with leukocytes traits are visualized in a compound network in Figure 1. These metabolites are involved in different metabolic pathways, including vitamin H (biotin) metabolism, vitamin B9 (folate) metabolism, urea cycle and metabolism of arginine, proline, glutamate, aspartate and asparagine, histidine metabolism, glycine, serine, alanine and threonine metabolism.

Estimates of genetic correlations between metabolites and RBC and platelet traits are shown in Table 2. L-proline was highly genetically positively correlated with HGB (*p* < 0.05) and HCT (*p* < 0.05). A high negative genetic correlation was observed for L-tyrosine with MCV (*p* < 0.05) and MCH (*p* < 0.05). In addition, L-glycine was positively correlated at the genetic level with MCH (*p* < 0.05). L-alanine (-*p* < 0.05) and L-alpha-aminobutyric acid (*p* < 0.05) were negatively correlated with RDW. Furthermore L-alanine was positively correlated with PLT (*p* < 0.05). Finally, the amino acid L-methionine was negatively correlated with MPV (*p* < 0.05). Basophils, RBC, platelets, MCHC, and MCV were not genetically correlated with any of the metabolites. The genetic correlation between metabolites that are involved in the same pathway are shown in Table 3 (below diagonal). The highest significant positive correlation was observed between oxoglutarate and L-glutamic acid followed by the correlation between L-serine and L-glycine.

**TABLE 3:** Estimates and standard errors (SE) of genetic correlations (below diagonal) and phenotypic correlation (above diagonal) between metabolites involved in the same pathway.

Metabolites	Betaine	Dimethylglycine	Glycine	L-glutamic acid	L-Serine	Oxoglutarate	L-Glutamine	Methionine
Betaine	1	0.40 (0.03)**	0.33 (0.04)*	−0.03 (0.03)	0.35 (0.03)**	0.01 (0.04)	−0.002 (0.04)	0.24 (0.04)**
Dimethylglycine	0.28 (0.14)	1	0.09 (0.04)	-	0.13 (0.04)*	−0.06 (0.04)	−0.12 (0.04)**	0.029 (0.03)
L-glycine	0.19 (0.19)	0.16 (0.21)	1	0.20 (0.03)***	-	0.25 (0.05)***	0.18 (0.03)***	0.01 (0.04)
L-glutamic acid	−0.14 (0.20)	−0.22 (0.26)	−0.007 (0.18)	1	0.27 (0.03)**	0.69 (0.02)***	0.42 (0.034)***	0.09 (0.04)
L-serine	−0.02 (0.47)	0.21 (0.33)	0.76 (0.52)***	0.14 (0.29)	1	0.27 (0.03)	0.22 (0.04)***	0.19 (0.04)*
Oxoglutarate	0.002 (0.22)	−0.18 (0.20)	0.22 (0.18)	0.79 (0.09)***	0.39 (0.34)	1	0.27 (0.03)***	0.05 (0.04)
L-glutamine	−0.37 (0.20)***	**−0.28 (0.17**)^**x**^	−0.23 (0.21)	0.2 (0.16)	−0.16 (0.34)	0.38 (0.18)*	1	0.33 (0.03)***
L-methionine	0.07 (2.25)	0.07 (0.22)	−0.59 (0.29)**	0.13 (0.40)	−0.46 (1.43)	0.11 (0.30)	0.3 (0.26)	1

Significance of the genetic and phenotypic correlations are highlighted in bold based on the likelihood ratio test and indicated as ***, **, *, ^X^, corresponding to *p* < 0.001, *p* < 0.01 and *p* ≤ 0.05, and 0.05 < *p* < 0.10 respectively; “-” indicates not estimable.

### GWAS

GWAS was performed for L-alpha aminobutyric acid, L-aspartate, L-asparagine, L-glutamic acid, L-glutamine, L-glycine, L-histidine, L-lysine, L-methionine, L- ornithine, L-serine, betaine, creatinine, dimethylglycine, hypoxanthine, isobutyric acid, oxoglutarate, 2-hydroxybutyrate and 3-methyl-2 oxovaleric acid because in the present study they were estimated to be genetically correlated with CBC, and in a previous study they were genetically correlated with resilience, or production traits (Dervishi et al., 2021).

For L-glutamic acid, the five most important windows were located on SSC14 and explained 7% of the genetic variance (Table 4). In addition, for L-tyrosine two windows on SSC1 explained 1.4% and 2.56% of the genetic variance. For L-asparagine, the most important window explained 1.1% of the genetic variance was found on SSC6. For creatinine a window on SSC12 explained 1.2% of the genetic variance and for hypoxanthine a window on SSC4 explained 1.2% of the genetic variance. One window on SSC6 explained 1.1% of the genetic variance for L-aspartate. Finally, one window on SSC2 explained 1.1% of the genetic variance of isobutyric acid.

**TABLE 4:** Chromosome position (in basepairs) and the proportion of additive genetic variance explained for each metabolite and genes located in the region.

Metabolite	Chromosome	Start (bp)	End (bp)	Variance explained by adjacent SNPs	Gene name
L-alanine	1	77,666,621	78,166,009	0.7	*FYN, U6, CCN6, TUBE1*
	13	148,254,310	148,753,746	0.6	*NECTIN3*
	14	98,696,648	99,196,606	0.54	*PRKG1, A1CF, N-acylsphingosine amidohydrolase 2*
L-asparagine	1	134,389,442	134,886,597	0.71	*CDIN1*
	6	63,321,290	63,818,847	0.65	*NOC2L, KLHL17, PLEKHN1, HES4, ISG15, AGRN, RNF223, ssc-mir-200b, ssc-mir-429, TNFRSF4, SDF4, B3GALT6, C1QTNF12, UBE2J2, ACAP3, PUSL1, INTS11, TAS1R3, DVL1, MXRA8, AURKAIP1, CCNL2, MRPL20, TMEM88B, VWA1, ATAD3A, TMEM240, SSU72, MIB2, MMP23B*
	6	65,142,458	66,987,107	2.49	*TPRG1L, WRAP73, TP73, CCDC27, SMIM1, LRRC47, CEP104, ssc-mir-2320, DFFB, C1orf174, ssc-mir-4331-1, AJAP1, NPHP4, KCNAB2*
L-aspartate	6	24,433,478	24,931,615	1.06	*U2*
	6	25,256,424	25,751,462	0.63	*None*
	6	70,267,482	70,764,647	0.88	*LZIC, NMNAT1, RBP7, UBE4B, KIF1B, U6, PGD, CENPS*
	14	43,982,025	44,481,714	0.82	*SEZ6L, ASPHD2, HPS4, SRRD, TFIP11, TPST2, CRYBB1, CRYBA4*
	14	106,941,420	107,439,174	0.61	*SORBS1, ALDH18A1, TCTN3, ENTPD1, CC2D2B*
	14	107,490,232	107,987,938	0.96	*CC2D2B, CCNJ, ZNF518A, BLNK, DNTT, OPALIN*
L-glutamic Acid	14	44,945,208	45,444,193	1.10	*MN1, PITPNB, TTC28*
	14	46,222,942	46,720,805	1.54	*ZNRF3, C22orf31, KREMEN1, EMID1, RHBDD3, EWSR1, GAS2L1, RASL10A, AP1B1, SNORD125, NEFH, THOC5, NIPSNAP1, NF2*
	14	46,863,516	47,363,323	1.36	*MTMR3, HORMAD2, LIF, OSM, CASTOR1, TBC1D10A, SF3A1, RNF215*
	14	47,569,039	48,065,569	2.06	*OSBP2, MORC2, TUG1, SMTN, SELENOM, INPP5J, PLA2G3, RNF185, LIMK2, PIK3IP1*
	14	49,337,114	49,836,497	0.89	*SPECC1L, ADORA2A, UPB1, GUCD1, LRRC75B, GGT5, SUSD2, CABIN1, DDT, GSTT4*
L-glutamine	12	9,686,276	10,186,144	0.57	*None*
	14	47,589,083	48,088,498	0.65	*OSBP2, MORC2, TUG1, SMTN, SELENOM, INPP5J, PLA2G3, RNF185, LIMK2, PIK3IP1, PATZ1*
	14	49,337,114	49,836,497	0.72	*SPECC1L, ADORA2A, UPB1, GUCD1, LRRC75B, GGT1, GGT5, SUSD2, CABIN1, DDT, GSTT4*
	14	107,490,232	107,987,938	0.54	*CC2D2B, CCNJ, ZNF518A, BLNK, DNTT, OPALIN, TLL2, TM9SF3*
L-glycine	6	41,826,913	42,324,310	0.54	*ZNF507*
	7	85,729,010	86,228,917	0.82	*RGMA, CHD2*
	13	67,207,607	67,707,171	0.53	*HRH1, ATG7, VGLL4*
L-histidine	2	134,063,036	134,562,065	0.53	*FNIP1, MEIKIN, ACSL6, CSF2, P4HA2, PDLIM4*
	6	66,977,105	67,476,230	0.6	*KCNAB2, CHD5, RPL22, RNF207, ICMT, HES3, GPR153, ACOT7, HES2, ESPN, TNFRSF25, PLEKHG5, NOL9, TAS1R1, ZBTB48, KLHL21, PHF13, THAP3, DNAJC11*
	7	49,996,822	50,495,753	0.69	*IL16, STARD5, TMC3*
	10	8,877,853	9,376,716	0.58	*LYPLAL1*
	14	9,447,641	9,946,090	0.6	*GNRH1, KCTD9, CDCA2, EBF2*
L-lysine	1	73,972,391	74,471,148	1.02	*SEC63, OSTM1, NR2E1, SNX3, AFG1L*
	1	74,474,926	74,974,129	0.58	*AFG1L, FOXO3, ARMC2*
	15	107,259,807	107,753,851	0.74	*CTLA4, ICOS*
	16	25,159,487	25,656,089	0.63	*None*
L-methionine	4	72,410,298	72,909,455	0.51	*CHD7, RAB2A*
	14	99,323,188	99,821,978	0.54	*MINPP1, PAPSS2, ATAD1*
L-ornithine	1	73,972,391	74,471,148	0.92	*SEC63, OSTM1, NR2E1, SNX3, AFG1L*
	1	8,554,166	9,054,053	0.89	*DYNLT1, TMEM181, GTF2H5, SERAC1, SYNJ2*
	3	9,162,389	9,658,871	0.56	*collagen type XXVI alpha 1 chain, MYL10, CUX1*
L-proline	1	73,972,391	74,471,148	0.58	*SEC63, OSTM1, NR2E1, SNX3, AFG1L*
	1	116,533,603	117,031,491	0.57	*RAB27A, RSL24D1*
	6	65,802,953	66,302,936	0.58	*AJAP1*
L-Serine	6	65,676,029	66,175,375	0.65	*AJAP1*
	14	46,499,201	46,998,737	0.54	*AP1B1, NEFH, THOC5, NIPSNAP1, NF2, CABP7, ZMAT5, UQCR10, ASCC2, MTMR3*
	14	47,569,039	48,065,569	0.62	*OSBP2, MORC2, TUG1, SMTN, SELENOM, INPP5J, PLA2G3, RNF185, LIMK2, PIK3IP1*
	17	31,756,334	32,255,590	0.7	*RNF24, PANK2, ssc-mir-103-2, AP5S1, CDC25B, CENPB, SPEF1, C20orf27, HSPA12B, HSPA12B, SIGLEC1, ADAM33, GFRA4, ATRN, U6*
L-tyrosine	1	73,972,391	74,471,148	1.16	*SEC63, OSTM1, NR2E1, SNX3, AFG1L*
	1	74,642,280	75,141,490	1.4	*FOXO3, ARMC2, SESN1*
	1	75,293,381	75,793,269	0.65	*CD164, PPIL6, SMPD2, MICAL1, ZBTB24, FIG4*
	1	80,371,029	80,869,342	0.68	*None*
	3	28,372,233	28,869,026	0.55	*ABCC1, BFAR, PARN*
Betaine	1	37,056,251	37,556,077	0.58	*NCOA7, HEY2*
	2	86,542,827	87,041,852	0.53	*TBCA, AP3B1*
	2	87,468,329	87,968,307	0.53	*ARSB, DMGDH, BHMT2, BHMT*
	6	82,101,618	82,599,859	0.57	*STPG1, NIPAL3, RCAN3, U6, SRRM1, CLIC4, RUNX3*
	15	124,184,229	124,681,509	0.69	*PAX3, SGPP2, MOGAT1*
	17	26,471,906	26,969,482	0.82	*PET117, KAT14, U6, ZNF133, DZANK1, POLR3F, RBBP9, SEC23B, SMIM26, DTD1*
Creatinine	2	149,629,454	150,127,355	0.74	*SPINK9, FBXO38, HTR4, ADRB2*
	12	2,562,992	3,060,017	1.15	*CBX8, CBX2, ENPP7, U6*
Dimethylglycine	2	85,842,752	86,340,606	0.51	*AGGF1, PDE8B, SNORA47*
	2	86,505,377	87,003,259	0.93	*OTP, TBCA, AP3B1*
	2	87,468,329	87,968,307	0.60	*ARSB, DMGDH, BHMT2, BHMT*
	6	82,101,618	82,599,859	0.64	*STPG1, NIPAL3, RCAN3, U6, SRRM1, CLIC4, RUNX3*
	15	129,051,568	129,551,348	0.86	*SCYGR5, SCYGR6, SCYGR8, C-C motif chemokine ligand 20, DAW1, SPHKAP, U6*
Hypoxanthine	3	10,293,739	10,789,778	0.70	*RHBDD2, CCL26, HIP1, NSUN5, TRIM50, FKBP6, FZD9, BAZ1B*
	4	129,471,881	129,968,024	1.18	*CLCA1, CLCA2, ODF2L, COL24A1*
	4	130,191,423	130,690,509	0.76	*COL24A1, ZNHIT6, CCN1, DDAH1, BCL10, C1orf52*
	5	69,586,920	70,086,295	0.60	*CECR2, BCL2L13, BID, MICAL3*
	17	31,589,919	32,084,533	0.66	*SMOX, U6, RNF24, PANK2, ssc-mir-103-2, AP5S1, CDC25B, CENPB, SPEF1, C20orf27, HSPA12B, SIGLEC1, ADAM33, GFRA4, ATRN*
	17	32,123,118	32,620,235	1.14	*ATRN, U6, DNAAF9, SLC4A11, ITPA, DDRGK1, LZTS3, FASTKD5, UBOX5, AVP, OXT*
Isobutyric acid	2	149,658,663	150,158,529	1.10	*FBXO38, HTR4*
	4	129,471,881	129,968,024	0.52	*CLCA1*
	12	2,562,992	3,060,017	0.59	*CBX8, CBX2, ENPP7, U6*
L-alpha-aminobutyric acid	14	107,490,232	107,987,938	0.62	*CC2D2B, CCNJ, ZNF518A, BLNK, DNTT, OPALIN*
	14	110,517,873	111,017,235	0.54	*CNNM1, GOT1, NKX2-3, SLC25A28, ENTPD7, ENTPD7, COX15, CUTC, ABCC2*
Oxoglutarate	1	25,502,333	26,001,840	0.83	*REPS1, ECT2L, CCDC28A, NHSL1, SNORA70, heme binding protein 2*
	1	24,945,041	25,443,993	0.61	*U6, CITED2, TXLNB, HECA, ABRACL*
	2	134,496,051	134,994,766	0.64	*P4HA2, PDLIM4, SLC22A4, SLC22A5, RAD50, IRF1, IL5, KIF3A, IL13, IL4*
	14	46,622,301	47,118,015	0.58	*THOC5, NIPSNAP1, NF2, CABP7, ZMAT5, UQCR10, ASCC2, MTMR3, HORMAD2*
	14	47,569,039	48,065,569	0.85	*OSBP2, MORC2, TUG1, SMTN, SELENOM, INPP5J, PLA2G3, RNF185, LIMK2, PIK3IP1*
	14	49,337,114	49,836,497	0.56	*SPECC1L, ADORA2A, UPB1, GUCD1, LRRC75B, GGT5, SUSD2, CABIN1, DDT, GSTT4*
2-hydroxybutyrate	7	85,799,632	86,298,534	0.55	*RGMA, CHD2*
3-methyl-2-oxovaleric acid	1	4,465,007	4,963,404	0.95	*QKI*
	13	26,247,606	26,746,605	0.54	*CCDC13, HIGD1A, ACKR2, CYP8B1, ZNF662, POMGNT2, SNRK, ANO10*
	15	122,986,770	123,486,192	0.53	*EPHA4*
	16	18,909,569	19,408,454	0.84	*NPR3*

The results of GWAS showed that some windows overlapped for some of the metabolites. For example, the window on SSC14 (49,337,114–49,836,497 bp) explained some of the genetic variance for both L-glutamic acid and glutamine. In this window a total of 10 genes were found (*SPECC1L, ADORA2A, UPB1, GUCD1, LRRC75B, GGT5, SUSD2, CABIN1, DDT, GSTT4*). Another window on SSC2 (87,468,329–87,968,307 bp) was found to explain genetic variance for both betaine and dimethylglycine. Four genes were located in this window (*ARSB, DMGDH, BHMT2* and *BHMT*)*.* In addition, one window on SSC1 (73,972,391–74,471,148 bp) was found to explain some of percentages of the genetic variance for L-ornithine, L-proline, and L-tyrosine. A total of 5 genes were found in this region (*SEC63, OSTM1, NR2E1, SNX3* and *AFG1L*)*.*

**FIGURE 2:**
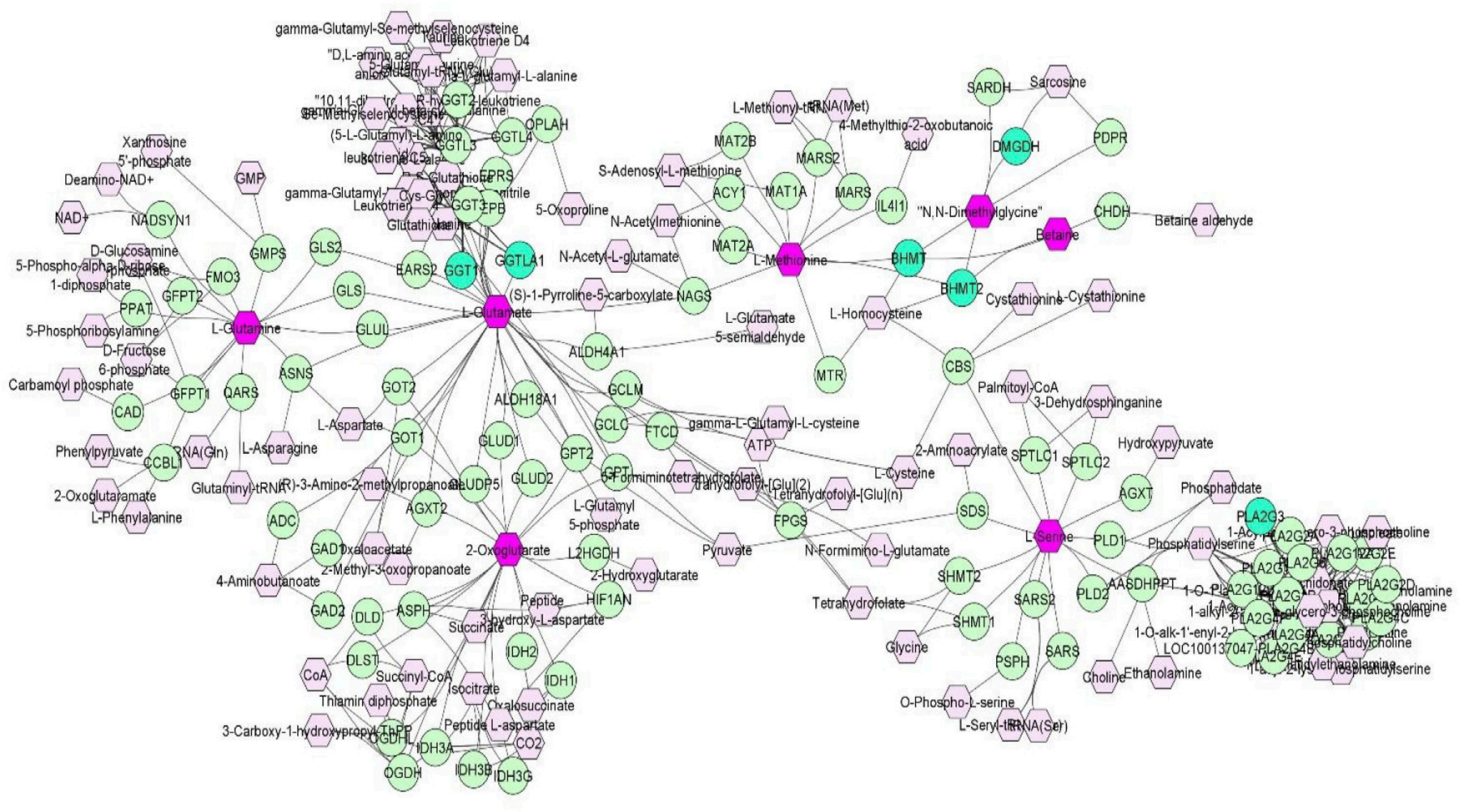
A compound-gene network of metabolites that are significantly (*p* < 0.05) genetically correlated with traits of interest (NEU, EOS, MCH, ADG and number of veterinary treatments). Input metabolites are shown in dark purple hexagonal and input genes are shown in bright green circles. Chemical reactions are represented as edges.

Discovery of common genomic regions for different metabolites led us to further investigate some genes that control the genetic variance of different metabolites. Thus, a compound-gene network of metabolites that had significant (*p* < 0.05) genetic correlations with traits of interest (example: neutrophils, EOS, MCH, ADG and number of veterinary treatments), and for which had overlapping genomic windows that explained genetic variance. Figure 2 shows the input metabolites (in dark purple color) and chemical reactions are represented as edges. The genes that are associated with these metabolites are displayed in bright green. For example, Figure 2 shows that betaine and dimethylglycine metabolism are interconnected. Genes located in same window on SSC2, (*DMGDH, BHMT2, BHMT*), are associated with both metabolites, suggesting pleiotropic effects of genes on multiple metabolites.

## Discussion

To our best knowledge, this is the first study reporting genetic correlation between metabolites and CBC traits in healthy young pigs. The aims of the present study were to estimate 1) genetic correlations between thirty-three heritable metabolites with CBC traits in healthy young pigs, and 2) identify genomic regions controlling the genetic variance of metabolites that were genetically correlated with CBC and production and disease resilience traits. Identification of genetic predictors/markers of resilience and production traits under disease challenge by using samples from healthy pigs is necessary for genetic selection programs in high health nucleus farms. This study contributes to our understanding of the relationship of metabolites and immune response cells in pigs. The results of phenotypic and genetic correlations increase our understanding of the crosstalk between metabolites and immune cells in healthy animals, which might help design nutritional and selection strategies to improve resilience. Meanwhile, it is possible to estimate the genetic correlations between different traits, it is often not clear what molecular processes contributes to these genetic correlations. Thus, this study deepens our understanding to what contributes to the genetic correlations at a molecular level in young healthy pigs.

### Phenotypic and genetic correlations

Phenotypic correlations of metabolites with CBC traits were generally very low. D-glucose had negative phenotypic correlation estimates with NEU, LYM, MONO, EOS, BASO, and MPV, while lactic acid was phenotypically positively correlated with WBC, NEU, LYM, BASO, EOS, RBC, HGB and MPV. Lactic acid is a hydroxycarboxylic acid and is mainly produced in muscle cells and red blood cells during anaerobic glycolysis (Connor et al., 1983). During glycolysis, glucose is metabolized into pyruvate, ATP, and NADHs. In the presence of oxygen, pyruvate is converted to acetyl-CoA in the tricarboxylic acid (TCA) cycle. Under oxygen-deprived conditions, pyruvate is reduced to lactate. However, the conversion of pyruvate to lactate also occurs under aerobic conditions, under which glucose uptake increases and preferential production of lactic acid takes place and it is known as the “Warburg effect” (Manca et al., 2021). In humans, increased levels of lactic acid are often used as biomarkers for various diseases, including autoimmune diseases, sepsis and neoplasia malignancy (Jansen et al., 2009; Ippolito et al., 2019). Lactic acid has been observed to be released by neutrophils in humans and mice (Stanfield and Germann, 2008; Kilkenny et al., 2010), acting as a critical regulator of neutrophil mobilization from the bone marrow. In ruminants, a greater concentration of lactic acid in blood is associated with ruminal acidosis (Hernández et al., 2014) and other health issues. In addition, lactic acid is reported to decrease platelet aggregation in horses and dogs (Lam et al., 2021; Lanier et al., 2022). Interestingly, our earlier work on these same data showed that lactic acid concentration in healthy pigs was found to be heritable (Dervishi et al., 2021), however, in this study we did not find significant genetic correlation estimates between lactic acid and CBC traits. Nonetheless, the results of phenotypic correlation are consistent with the possible role of lactic acid in exerting immunomodulatory effects that regulate the inflammatory response (Hoque et al., 2014; Pucino et al., 2017; Manca et al., 2021).

Overall, estimates of genetic correlations between metabolites and CBC traits ranged from moderate to high. We found high negative genetic correlation between plasma L-lysine and NEU (−0.81 ± 0.4), which is in agreement with previous studies reporting that lysine can modulate the neutrophil metabolism (Hu et al., 2016). L-Lysine is an essential amino acid in protein biosynthesis. Pigs have a high requirement for lysine (Liao et al., 2015) and supplementation with lysine can improve muscle protein accretion in pigs (Wu et al., 2014). On the other hand, impaired immune function, and increase susceptibility to infectious diseases have been reported in animals receiving a diet deficient in lysine (Chen et al., 2003). In pigs, (Brooks et al., 1964) found that lysine supplementation resulted in reductions in white blood cell counts, gamma globulin levels, and sedimentation rates, and increases in hematocrit values, red cell counts, hemoglobin levels, total serum protein levels, and serum albumin levels. In rats, supplementation of lysine significantly reduced the neutrophil, lymphocyte counts, the tumor necrosis factor alpha (TNF-α), interleukin-8 (IL-8), and migration inhibitory factor (MIF) levels and protected against sepsis-induced chronic lung injury (Zhang et al., 2019). Lysine is found in high abundance in histones and lysine residues in the histones are accessible to several post translational modifications, including methylation and acetylation. Neutrophils are key participant in the innate immune response with a short half-life varying from 8–20 h and the regulation of neutrophil death rate is essential for maintaining hemostasis under physiological conditions (Perez- Figueroa et al., 2021). One of the defense mechanism of neutrophils is the formation of neutrophil extracellular traps (NETs; Brinkmann et al., 2004), which consist of DNA fibers associated with histones, enzymes from neutrophil granules and anti-microbial peptides which are released in the extracellular environment. The release of NETs is also a part of programmed cell-death process called NETosis. Lysine is important amino acid which is involved in histone modification process that take place during NETosis which will result in the formation of dispersed chromatin (Poli et al., 2021; Pérez-Figueroa et al., 2021). This molecular process might explain the negative genetic correlation between lysine and neutrophils. Our results suggest that L-lysine modulates neutrophil concentration and hence the immune response in pigs. The molecular aspect of it needs to be clarified and deserve further investigation.

We found that L-glutamine was genetically positively highly correlated with NEU and L-aspartate was positively correlated with MONO. Positive genetic correlation estimates were observed for the amino acids L-asparagine and L-glutamic acid with WBC. Lymphocyte concentration was negatively correlated with hypoxanthine and L-ornithine. Extensive research conducted in human and animal studies have demonstrated the role of amino acids in immune cell maturation, modulation, and function. For example, amino acids such as glutamic acid, glutamine, histidine, methionine, leucine, isoleucine, and valine are functional regulators of macrophages, dendritic cells, and T cells (Yoneda et al., 2009; McGaha et al., 2012; Wu, et al., 2012; Liu et al., 2017).

One of our most interesting results is the high positive genetic correlation between L-proline and hemoglobin concentration (0.94 ± 0.03). Using NMR spectroscopy and mutagenesis Gell et al. (2009) identified the importance of an evolutionary conserved proline residue in α-hemoglobin stabilizing protein. The results of genetic correlation between hemoglobin and proline may reflect the fact that proline is necessary for the structural reorganization of α-hemoglobin (Gell et al., 2009) and for the synthesis of iron related proteins such as hemoglobin, ferritin, and transferrin (Kitajima et al., 2003). Indeed, in rats, supplementation with proline enhanced a significant increase in the number of red blood cells and hemoglobin (Kitajima et al., 2003).

In addition, isobutyric acid was found to be positively genetically correlated with LYM concentration (0.69 ± 0.35) in healthy pigs. Previously, Dervishi et al. (2021), using the same pig population, reported that isobutyric acid in these pigs is heritable and negatively genetically correlated with residual feed intake under disease (RFI; −0.38). Furthermore, LYM concentration in young healthy pigs was found to be negatively genetically correlated with veterinary treatment rate under disease (Bai et al., 2020) in the same pig population. These results suggests that isobutyric acid might modulate both traits, LYM in young healthy pigs and RFI in pigs under disease conditions. Further research is necessary to investigate the relationship between isobutyric acid and LYM levels in blood of young healthy pigs, and RFI under disease.

Other metabolites such as 2-hydroxybutyrate, 3-methyl-2-oxovaleric acid and L-alpha aminobutyric acid were estimated to have positive genetic correlations with LYM and EOS concentration meanwhile dimethylglycine was negatively correlated with EOS. Furthermore, we found that the amino acids L-alanine and L-alpha-aminobutyric acid, also called homoalanine, are negatively genetically correlated with RDW. The red blood cell distribution width is a measure of size variability and heterogeneity of erythrocytes in the pheripheral blood and reflects the degree of anysocytosis. At present it is not clear why L-alpha aminobutyric and L-alanine are genetically correlated with RDW, but it is worth further investigation. For many other metabolites such as 2-hydroxybutyrate and 3-methyl-2-oxovaleric acid, literature on genetic correlations is lacking, making the interpretation of our results difficult.

### GWAS

We performed a GWAS for 22 metabolites in young healthy pigs that were genetically correlated either with production or resilience traits or with CBC traits and investigated the genomic regions that explained a sizeable proportion of the variance for each metabolite. GWAS with CBC traits, were previously reported by Bai et al. (2020). Interestingly, Bai et al. (2020) proposed Member RAS Oncogene Family (*RAB32*) located on SSC1 as a candidate gene for RBC concentration. In the present study we found Member RAS Oncogene Family (*RAB27A*), located on SSC1 as a candidate gene for L-proline. In addition, proline was found to be genetically correlated with HGB concentration and HCT. These results might suggest that member RAS oncogene family (RAB32 and RAB27A) and proline modulate RBC traits, however further functional studies are necessary to validate our results. In addition, Bai et al. (2020) proposed tubulin beta class VI coded by TUBB1 (tubulin beta 1 class VI) on SSC17 as candidate gene for mean platelet volume (MPV fL). In our study we found that TUBE1 (tubulin epsilon 1) on SSC1, explains a small percentage of genetic variation of L-alanine which is positively genetically correlated with platelet concentration (PLT 10^3^/μL) suggesting a possible role of L-alanine and tubulin superfamily in platelets concentration.

Furthermore, in our study we found that a window on SSC1 containing five genes (*SEC63*, *OSTM1, NR2E1, SNX3* and *AFG1L*), explained 1.02% of genetic variation of L-lysine concentration which was negatively genetically correlated with NEU concentration. Interestingly NR2E1 (Nuclear Receptor Subfamily 2, Group E, Member) functions as a repressor and activator of gene transcription (Corso-Díaz et al., 2016). As repressor of gene expression, NR2E1 interacts with co-repressor histone Lysine-specific demethylase 1 (LSD1) (Yokoyama et al., 2008). There is evidence that acetylation of lysine residues of other transcription factor such as C/EBPε is necessary for terminal neutrophil differentiation (Bartels et al., 2015). Our results suggest a possible epigenetic role of L-lysine and NR2E1 gene in modulating NEU concentration. However functional studies are necessary to validate our results.

Overall, the percentage of genetic variance explained by each window was small (<2%) for all metabolites. For L-glutamic acid, there were five important neighboring windows on SSC14 that together explained 6.9% of the genetic variance. Interestingly, we found overlapping windows on SSC14 that explained variance for L-glutamic acid, L-glutamine, L-serine, and oxoglutarate (example: 47,569,039–48,065,569 bp; 49,337,114–49,836,497 bp). These overlapping genomic windows might explain the positive genetic correlation between L-glutamic acid and oxoglutarate. Other overlapping windows were detected on SSC1 for L-lysine, L-ornithine, L-proline, and L-tyrosine (73,972,391–74,471,148 pb), on SSC2 for betaine and dimethylglycine (87,468,329–8768,307), on SSC6 for L-asparagine, L-proline, and L-serine (65,676,029–66,175,375 pb), and on SSC12 for creatinine and isobutyric acid (2,562,992–3060017 pb). These overlapping windows on SSC2 might contribute to the positive genetic correlation observed between betaine and dimethylglycine. These results suggest pleiotropic effects of loci on metabolite concentrations, i.e., that the same gene may control variation in more than one metabolite. Pleiotropy effects of loci on metabolites have been previously described in human studies (Smith et al., 2022). To further investigate, we attempted to integrate metabolites and genes in a single network (Figure 2). Indeed, we found that some of the genetic variation of metabolites such as L-glutamic acid, L-glutamine, oxoglutarate, dimethylglycine, betaine, and L-serine were explained by the same loci. For example, on SSC14 the window 47,569,039–48,065,569 pb contains *phospholipase A2 group III* (PLA2G3)*.* PLA2G3 is involved in L-serine metabolism and lipid metabolism. It catalyzes the calcium-dependent hydrolysis of the sn-2 acyl bond of phospholipids to release arachidonic acid and lysophospholipids. Phosphatidylserine is a phospholipid that consists of two fatty acids attached in ester linkage to the first and second carbon of glycerol and serine attached through a phosphodiester linkage to the third carbon of the glycerol (Nelson and Cox, 2008). In a previous study on these same pigs, Dervishi et al. (2021) reported that L-serine was positively genetically correlated with average daily gain in the quarantine nursery (0.54). This might suggest *PLA2G3* as a candidate gene for ADG in healthy young pigs. Additionally, the window 87,468,329–87,968,307 pb on SSC2 includes *arylsulfatase B* (ARSB), *dimethylglycine dehydrogenase* (DMGDH), *betaine-homocysteine S-methyltransferase* (BHMT), and *betaine-homocysteine S-methyltransferase 2* (BHMT2). DMGDH is involved in the catabolism of choline, catalyzing the oxidative demethylation of dimethylglycine to form sarcosine. BHMT and BHMT2 are methyl transferases. *BHMT* encodes a cytosolic enzyme that catalyzes the conversion of betaine and homocysteine to dimethylglycine and methionine, respectively. BHMT2 can catalyze the transfer of the methyl group from betaine to homocysteine to create methionine (Ganu et al., 2015). The metabolism of dimethylglycine, of betaine, and of methionine are intertwined, as is shown in Figure 2, suggesting pleiotropic effect of these genes on metabolites. Previously, in pigs *DMGDH* has been associated with total weight of live neonates per litter (Wu et al., 2018) and *BHMT* has been associated with number of muscle fibers per unit area (Wimmers et al., 2006). Interestingly, using the same pig population, Dervishi et al. (2021) showed that betaine, dimethylglycine, and methionine concentrations in blood of young healthy pigs were genetically positively correlated with growth rate in the quarantine nursery. Here we found that, in blood of healthy young pigs, dimethylglycine was genetically negatively correlated with EOS and the amino acid L-methionine was negatively correlated with MPV, suggesting a genetic connection between immune response and growth. Our results suggest *PLA2G3, DMGDH*, *BHMT,* and *BHMT2* as candidate genes for variation in L-serine, dimethylglycine, betaine, and L-methionine concentration. Furthermore, serine, dimethylglycine, betaine, and L-methionine might be candidate metabolites to improve nursery growth rate of young healthy pigs. Future Mendelian randomization analysis and/or functional experiments should be performed in order to confirm that the metabolite mediates the effect of the SNP/gene on the phenotype and to establish a causal relationship.

We previously reported that isobutyric acid concentration in blood of young healthy pigs is negatively genetically correlated with RFI under disease challenge, suggesting that young healthy pigs that have higher plasma isobutyric acid content genetically have lower RFI under disease conditions (Dervishi et al., 2021). The GWAS for isobutyric acid showed that the most important windows on SSC2 explained 1.1% of the genetic variance for isobutyric acid. Two genes have been annotated for this window; *F-box protein 38* (FBXO38) and *5-hydroxytryptamine receptor 4* (HTR4). *5-hydroxytryptamine receptor 4* is a member of the family of serotonin receptors, which are G protein coupled receptors that stimulate cAMP production in response to serotonin (5-hydroxytryptamine). Interestingly Yao et al. (2013) identified a significant SNP in *HTR4* to be associated with RFI in dairy cattle using the Random Forests (RF) algorithm. In addition, GWAS performed by Manca et al. (2021) suggested *HTR4* as a candidate gene for residual concentrate intake (RCI) in dairy cattle. In pigs there is no evidence in the literature to connect isobutyric acid with *HTR4* and RFI, making interpretation of our results challenging. However, 5-hydroxytryptamine receptor 2B, (*HTR2B*), which belongs to the same family of serotonin receptors as *HTR4,* was proposed as functional candidate gene for feed conversion ratio (FCR) in pigs (Horodyska et al., 2017). We think that further research is necessary to elucidate the relationship between isobutyric acid, *HTR4* and RFI.

## Conclusion

Phenotypic correlation estimates of plasma metabolites levels with CBC traits in young healthy pigs were generally low. Lactic acid might exert immunomodulatory effects that regulate the inflammatory response in pigs, which deserves further research and validation. This study showed significant genetic correlation estimates between metabolites and CBC traits in blood of young healthy pigs, demonstrating a potential role of metabolites in modulating the immune system. We identified candidate genes for part of the genetic variation of plasma metabolite concentrations. The GWAS demonstrated that the regulation of metabolites concentration is polygenic with no individual region, explaining a large proportion of the total genetic variation. Results of GWAS suggest that *DMGDH*, *BHMT,* and *BHMT2* are candidate genes for dimethylglycine, betaine and L-methionine concentration in blood of young healthy pigs, while dimethylglycine, betaine, and L-methionine are candidate metabolites to improve growth rate of young healthy pigs.

This study contributes to understanding the relationship of metabolites and immune response cells in pigs and can offer insights for human physiology and immune response, however replication studies and validation of our results in human samples are necessary.

## PigGen Canada Members

ALPHAGENE (2200, Pratte Avenue, Saint-Hyacinthe QC J2S 4B6 Canada), Hypor Canada (Spoorstraat 69, 5831CK, Boxmeer, Netherlands), Topigs Canada (20 South Landing, Unit 1, Oak Bluff, MB. R4G 0C4, Canada), Alliance Genetics (PO Box 24039 Edward RPO 107 Edward St. St. Thomas, ON N5P 1Y0, Canada), Fast Genetics (8—4003 Millar Avenue, Saskatoon, SK S7K 2K6, Canada), DNA Genetics (4438 Old Mill Court, Columbus, NE 68601, United States), Genesus Genetics (101 2nd St, Oakville, MB R0H 0Y0, Canada).

## Data Availability

The data analyzed in this study is subject to the following licenses/restrictions: The data analyzed in this study are not publicly available, because the data were generated on samples from commercially owned animals, however, they can be made available by the corresponding author on reasonable request. Requests to access these datasets should be directed to plastow@ualberta.ca.

## Supplementary material

SUPPLEMENTARY FIGURE S1 Time line and collected phenotypes during natural polymicrobial disease challenge model.

SUPPLEMENTARY FIGURE S2 A representative of NMR spectrum with assignments. represents full spectrum; **(B–D)** are the partial zoom views.

SUPPLEMENTARY FIGURE S3 Phenotypic correlation between metabolites and complete blood count traits.

SUPPLEMENTARY FIGURE S4 Scatter plot showing the negative phenotypic correlations between D-glucose, pyruvic acid and CBC traits.
