# Supplementary figures and images for: GWAS and genetic and phenotypic correlations of plasma metabolites with complete blood count traits in healthy young pigs reveal implications for pig immune response
Source: Front Mol Biosci. 2023 Mar 13;10:1140375. doi: 10.3389/fmolb.2023.1140375 (PMC10034349; doi:10.3389/fmolb.2023.1140375)

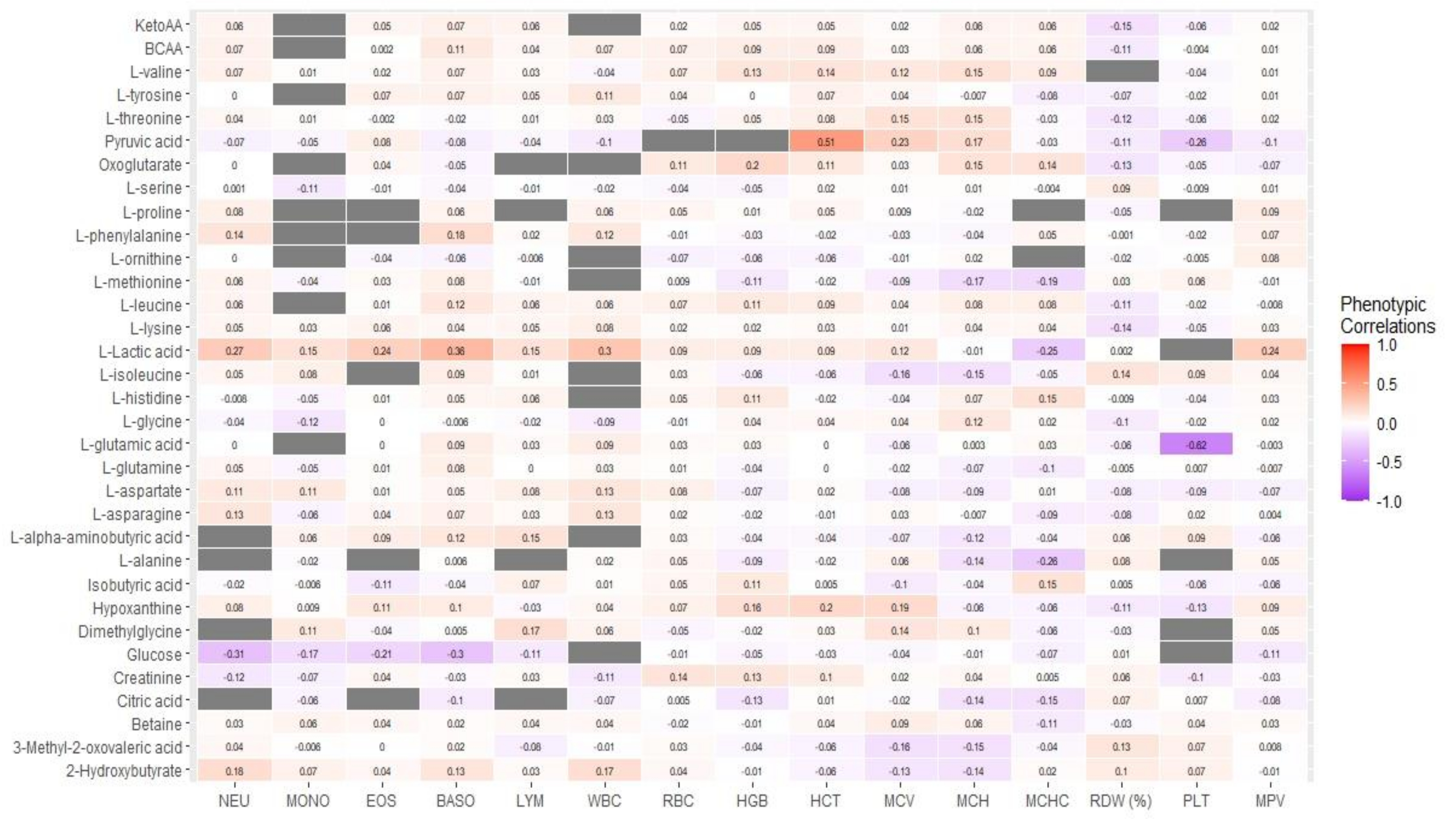

Supplement: Supplementary file 2 [file Image3.JPEG]

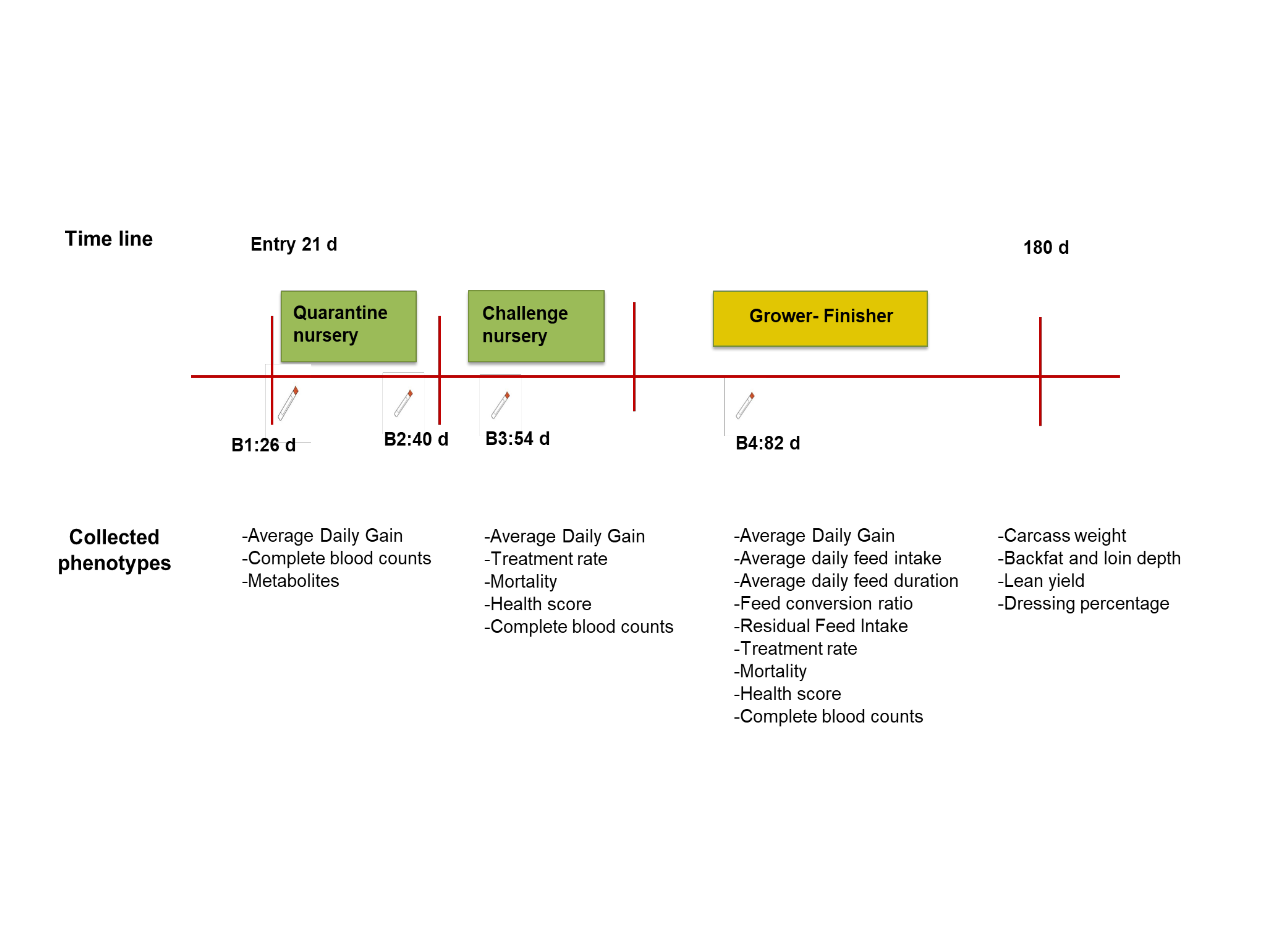

Supplement: Supplementary file 3 [file Image1.JPEG]

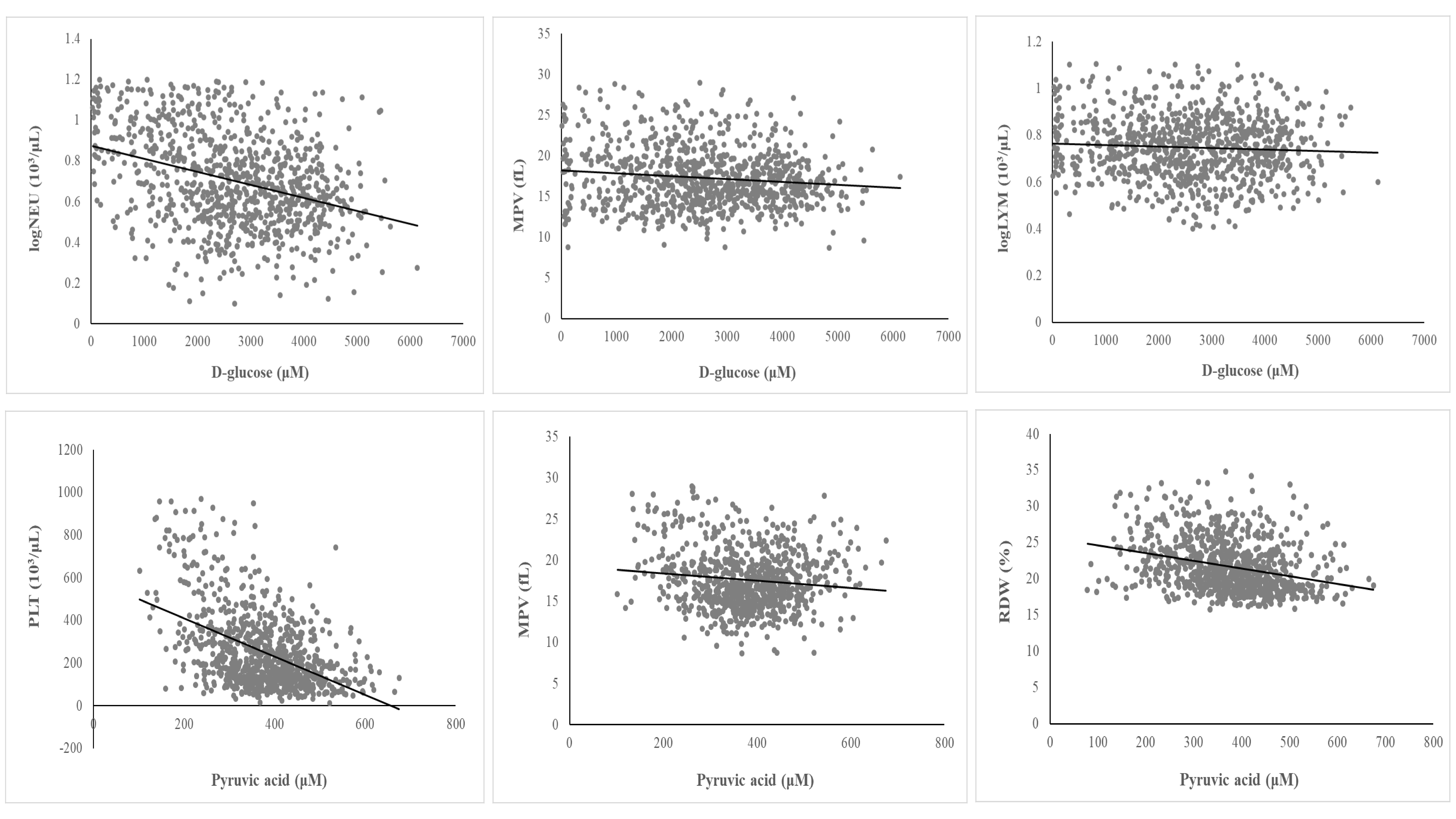

Supplement: Supplementary file 4 [file Image4.JPEG]

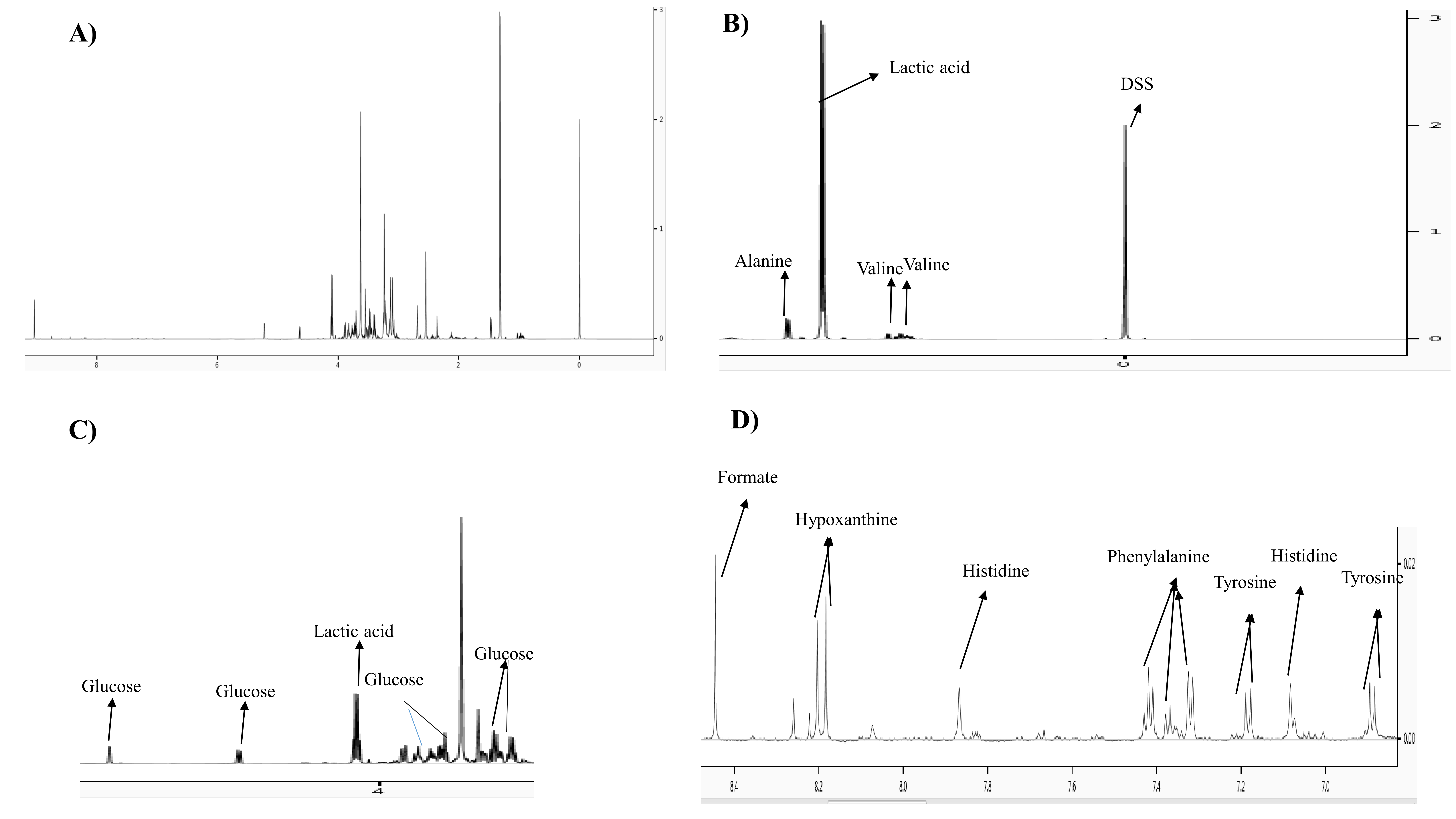

Supplement: Supplementary file 5 [file Image2.JPEG]
